# The Impact of Traditional Chinese Medicine QingreHuoxue Treatment and the Combination of Methotrexate and Hydroxychloroquine on the Radiological Progression of Active Rheumatoid Arthritis: A 52-Week Follow-Up of a Randomized Controlled Clinical Study

**DOI:** 10.1155/2022/5808400

**Published:** 2022-04-12

**Authors:** Rouman Zhang, Xiao-Po Tang, Jian Wang, Wei-Xiang Liu, Jian Liu, Yue Wang, Wei Liu, Yong-Fei Fang, Dong-Yi He, Ying Liu, Ming-Li Gao, Qing-Jun Wu, Zhen-Bin Li, Shi Chen, Qing-Chun Huang, Yan-Ming Xie, Jun-Li Zhang, Cai-Yun Zhou, Li Ma, Xin-Chang Wang, Quan Jiang, Xun Gong

**Affiliations:** ^1^Rheumatology, Guang'anmen Hospital China Academy of Chinese Medical Sciences, Beijing 100053, China; ^2^Rheumatology, University of Chinese Medicine, Hefei 230038, China; ^3^3 Rheumatology, Jiangsu Provincial Hospital of Traditional Chinese Medicine, Suzhou 215002, China; ^4^Rheumatology, The First Affiliated Hospital of Tianjin University of Traditional Chinese Medicine, Tianjin 300193, China; ^5^Rheumatology, The Southwest Hospital of AMU, Chongqing 400038, China; ^6^Rheumatology, Shanghai Guanghua Hospital of Integrated Traditional Chinese and Western Medicine, Shanghai 200052, China; ^7^Rheumatology, The Affiliated Hospital of Shandong University of Traditional Chinese Medicine, Jinan 250014, China; ^8^Rheumatology, The Affiliated Hospital of Liaoning University of Traditional Chinese Medicine, Shandong 250011, China; ^9^Rheumatology, Peking Union Medical College Hospital, Beijing 100730, China; ^10^Rheumatology, Bethune International Peace Hospital, Shijiazhuang 050000, China; ^11^Rheumatology, Peking University People's Hospital, Beijing 100044, China; ^12^Rheumatology, Guangdong Provincial Hospital of Traditional Chinese Medicine, Guangzhou 510220, China; ^13^Rheumatology, Institute of Basic Research in Clinical Medicine, China Academy of Chinese Medical Sciences, Beijing 100700, China; ^14^Rheumatology, The Fifth Hospital of Xi'an, Xi'an, China; ^15^Rheumatology, Xiyuan Hospital China Academy of Chinese Medical Sciences, Beijing, China; ^16^Rheumatology, China-Japan Friendship Hospital, Beijing 100029, China; ^17^Rheumatology, The Second Affiliated Hospital of Zhejiang University of Chinese Medicine, Hangzhou 310005, China

## Abstract

Traditional Chinese medicine (TCM) has been used successfully to treat rheumatoid arthritis (RA). QingreHuoxue treatment (QingreHuoxue decoction [QRHXD]/QingreHuoxue external preparation [QRHXEP]) is a Chinese medicine treatment for RA. To date, very few studies have compared the long-term effects of QRHXD with those of conventional disease-modifying antirheumatic drugs on RA disease activity and radiological progression. QRHXD delayed the radiological progression and showed long-term clinical efficacy of RA. In clinical experiments, the clinical evidence of delaying the radiological progression of RA patients was obtained. A portion of the patients who participated in the “Traditional Chinese Medicine QingreHuoxue Treatment vs. the Combination of Methotrexate and Hydroxychloroquine for Active Rheumatoid Arthritis” study were followed up for 52 weeks, and intention-to-treat (ITT) and compliance protocol (PP) analyses were used to collect and compare the clinical indicators and imaging data between baseline and week 52. Two radiologists who were blind to treatment scored the images independently. Of the 468 subjects, 141 completed the 52-week follow-up. There were no significant differences among the three groups: the traditional Chinese medicine comprehensive treatment group, the Western medicine treatment group, and the integrated traditional Chinese and Western medicine treatment group. There were no differences in the total Sharp score, joint space stenosis score, and joint erosion score at baseline or 52 weeks. In the comparison of the estimated annual radiographic progression (EARP) and the actual annual Sharp total score changes among the three groups, the actual changes were much lower than the EARP at baseline. The radiological progress in all three groups was well controlled. Results of the ITT and PP data sets showed that the disease activity score 28 level of the three groups at 52 weeks was significantly lower than that at baseline. During the 52-week treatment period, the clearance of heat and promotion of blood circulation controlled disease activity and delayed the radiological progress of active RA.

## 1. Introduction

Rheumatoid arthritis (RA) is an autoimmune disease with erosive arthritis as the main clinical manifestation [1]. Data have shown that approximately 90% of RA patients experience bone erosion within 2 years of the onset of disease, which eventually leads to joint deformity and disability [2, 3]. A long-term clinical study by Scott et al. [4] indicated that the X-ray damage of RA is closely related to the level of disability, which increases with longer disease duration. The continuous progression of RA directly causes the loss of function and even disability in patients.

RA with moderate to severe activity results in bone destruction, disease activity, and bone erosion are the main clinical focus. How to effectively control RA synovial inflammation, reduce disease activity, and delay bone destruction are the primary problems addressed in clinical research. In recent years, a considerable number of RA patients have benefited under the guidance of advanced treatment concepts, such as early-stage standardized treatments, standard treatments based on the single or combined use of disease-modifying antirheumatic drugs (DMARDs), and the use of new biological agents. However, DMARDs and biological agents still exhibit problems such as serious adverse reactions and the significantly increased risk of lung infection and tuberculosis infection [5–8]. Moreover, some patients who achieve disease remission still exhibit continuous progression based on imaging [9]. The treatment of RA bone destruction is a primary, yet challenging, focus of clinical work. Traditional Chinese medicines (TCMs) may be particularly suitable for the treatment of RA. Clinical trials have demonstrated that TCMs can effectively reduce the disease activity of RA, increase the rate of RA disease remission, and effectively delay the progression of RA.

To further evaluate the role of TCMs in the treatment of RA by comparing its effects with a combination treatment of methotrexate (MTX) and hydroxychloroquine (HCQ), we recently conducted a clinical study named “Traditional Chinese Medicine QingreHuoxue Treatment vs. the Combination of MTX and Hydroxychloroquine for Active RA” [10]. In this multicenter, double-blind, randomized controlled trial (RCT), 468 Chinese patients with active RA (disease activity score [DAS] 28 > 3.2) were treated with QingreHuoxue decoction (QRHXD/QRHXEP) (the TCM group), MTX plus HCQ (the Western medicine [WM] group), or both (integrative medicine [IM] group). QRHXD/QRHXEP was effective in alleviating symptoms of active RA, albeit to a lesser degree than conventional synthetic DMARDs (csDMARDs), with fewer side effects. These results provide evidence that QRHXD can be used as a kind of adjuvant of csDMARDs in the treatment of RA.

Our current disease management goal for RA patients is to not only control disease activity for a long term but also reduce long-term-related joint damage. In clinical research, in addition to the evaluation of disease activity, such as by the DAS28 and American College of Rheumatology (ACR) 20% response (ACR20), 50% response (ACR50), and 70% response (ACR70) compliance rates, the prevention of progression on imaging and the reduction of joint damage are also important outcomes for determining the long-term therapeutic effect and are recommended as RA evaluation indices of patients' overall functional status [11]. Our study directly addressed the main difficulty in the treatment of RA: bone destruction. The Sharp score was used as the main efficacy indicator. A 24-week clinical study that focused on the treatment of RA with QingreHuoxue treatment was conducted. After the trial was completed, we continued to follow up the patients and conducted an observational efficacy comparison study. After its termination, the patients continued to be followed up for 52 weeks and monitored disease activity. Patients' radiological images and disease activity were collected at week 52 to determine whether the same level of efficacy observed during the previous 24 weeks was sustained. This observational curative effect real-world comparison study of RA patients allowed the evaluation of the long-term effects of QingreHuoxue treatment, the integrated traditional Chinese and Western medicine, and Western medicine on the radiological progression and disease activity of RA. The study aimed to provide effective interventions for the treatment of RA and obtain high-level clinical evidence demonstrating that the comprehensive program of clearing heat and promoting blood circulation delays the process of RA bone destruction.

## 2. Materials and Methods

The clinical study “Traditional Chinese Medicine QingreHuoxue Treatment vs. the Combination of MTX and Hydroxychloroquine for Active RA” was designed as a 24-week, multicenter, double-blind, RCT. Following the trial, researchers followed up subjects for 52 weeks and subsequently adopted an observational efficacy comparison study design to review the radiographs of patients' hands and wrists to assess the progression of joint bone destruction. The primary endpoint of this study is the Sharp scoring system revised by van der Heijde at 52 weeks.

### 2.1. Patients

At the time of enrollment, patients who met the conditions of the trial also met the following criteria: (1) met RA classification criteria revised by the American Academy of Rheumatology (ARA) in 1987; (2) aged 18–65 years; (3) met TCM syndrome of damp-heat blockage and blood stasis blocking the collaterals; (4) had a DAS28 score >3.2; (5) patients were taking csDMARDs for at least 3 months at a stable dose and continued the same treatment for the duration of the present study; and (6) were willing to be followed up long term and provide 52-week hand radiology imaging data following the 24-week follow-up. Patients who completed the 24 weeks of the original study continued to be followed up for 52 weeks. All patients provided written informed consent at enrollment.

### 2.2. Study Treatment

The program was approved by the Ethical Review Committee of Guang'anmen Hospital, China Academy of Chinese Medical Sciences (no. 2013EC122).

Initially, 468 research cases from 17 research centers across the country were randomized and divided into three groups: the TCM group, the WM group, and the IM group. The specific treatments administered were as follows—the TCM group: QingreHuoxue recipe granules + QingreHuoxue external application + MTX tablet simulator + hydroxychloroquine sulfate tablet simulator; the WM group: MTX tablets + hydroxychloroquine sulfate tablets + TCM oral placebo particles + external placebo; the IM group: QingreHuoxue recipe granules + QingreHuoxue external application + MTX tablets + hydroxychloroquine sulfate tablets. MTX was taken 12.5 mg once weekly, and HCQ was taken 200 mg twice daily. QRHXD was taken twice daily for 24 weeks (1 bag boiled in water for each dose).

MTX and HCQ tablets were fabricated from Shanghai Xinyi Pharmaceutical Co. (Shanghai, China) and Shanghai Zhongxi Pharmaceutical Co. (Shanghai, China), respectively, and were taken orally. TCM QingreHuoxue treatment for RA “damp-heat-stasis syndrome” with QRHXD and QRHXEP supplied by the Guang'anmen Hospital China Academy of Chinese Medical Sciences was used in this study. QRHXD was gotten into granules that were packaged in a tin foil bag by Sichuan New Green Pharmaceutical Technology Development Co. (Chengdu, China). QRHXEP was processed into a gel formulation and packaged in a plastic tube at Guang'anmen Hospital China Academy of Chinese Medical Sciences (batch no. 15011303).

QRHXD has 12 components including animal drug wugong (centipede [4 g]) and the botanical drugs species or TCM plant preparations tufuling (*Smilax glabra* Roxb [30 g]), yinhua (*Lonicera japonica* Thunb [30 g]), huangqi (*Astragalus mongholicus* [30 g]), chaocangzhu (bran-fried *Atractylodes chinensis* [15 g]), huangbo (*Phellodendron amurense* [9 g]), chishao (*Paeonia lactiflora* [15 g]), bixie (Dioscoreae hypoglaucae rhizoma [15 g]), danshen (*Salvia miltiorrhiza* [15 g]), ezhu (*Curcuma zedoaria* [9 g]), qingfengteng (*Sinomenium acutum* [15 g]), and fengfang (Nidus vespae [5 g]). The granules were packaged as 10 g bags.

**Figure 1:**
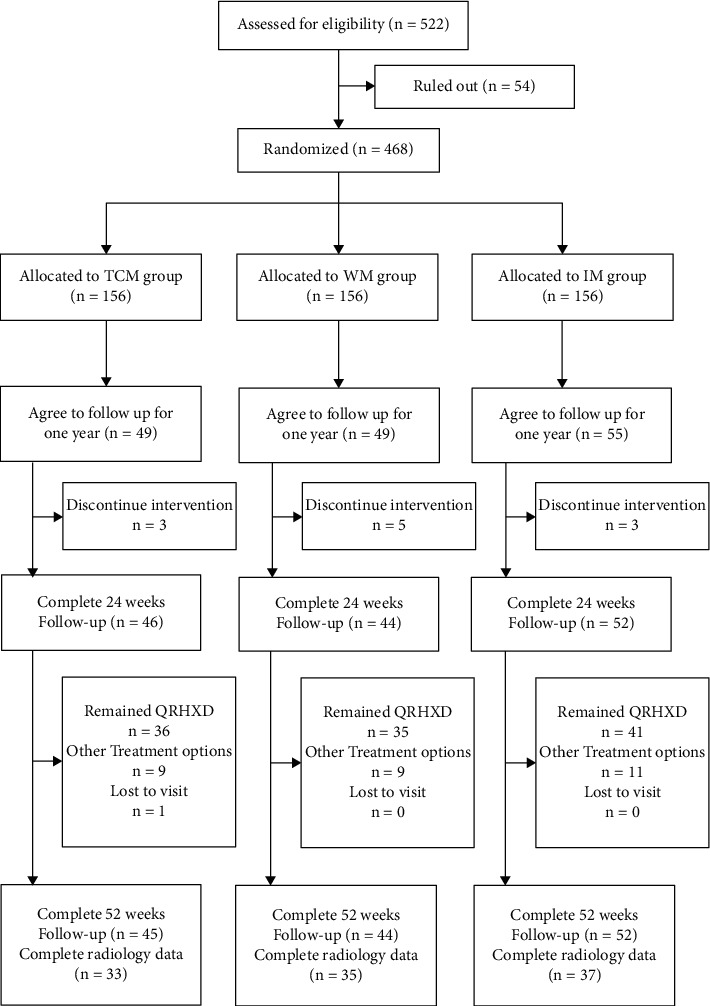
Study flowchart.

After the end of the study medication period (24 weeks), the follow-up period was up to 52 weeks, and the study was designed as an observational efficacy comparison study to achieve an open-label, case-control, and long-term follow-up study. During the 24–52 weeks, the treatment plan was adjusted and recorded according to the patient's condition. Most of the patients continued to use QingreHuoxue decoction, and some patients were changed to other treatment plans. Please refer to the study flowchart for details (Figure 1).

### 2.3. Outcomes and Measurements

The main indicator of this study was the evaluation of bone destruction. The primary endpoint of this study was the Sharp scoring system revised by van der Heijde (including joint erosion (JE) score, joint space reduction (JSN), revised total Sharpe score (TSS)) [12]. Subjects underwent radiological progression analysis at baseline and week 52, which involved frontal X-rays of both hands and wrists. Two radiologists read and analyzed the radiographic images according to the Sharp scoring system revised by van der Heijde. The radiologists had no knowledge of the treatment allocation, the chronology of radiographs, or patients' clinical responses. The joint erosion (JE) score and joint space narrowing (JSN) were added to calculate the revised total Sharp score (TSS) [13]. Differences between readers were assessed using the intraclass correlation coefficient, based on the status score, which ranged from 0.794 to 0.907. Secondary indicators were disease activity evaluation (DAS28), erythrocyte sedimentation rate, number of swollen joints, and number of tender joints. Safety evaluation indicators included the subjects' vital signs, such as blood pressure, respiration, and heart rate, as well as blood, urine, liver function, kidney function, and electrocardiograms of the subjects before and after the study at the baseline and 52 weeks.

### 2.4. Statistical Analysis

Intention-to-treat (ITT) and compliance protocol (PP) analyses were used to collect and compare the baseline and 52-week clinical indicators and radiographic data. Continuous data were presented as mean (SD), and categorical data were presented as numbers and/or percentages. A one-way ANOVA or Kruskal–Wallis rank test combined with the *t-*test or Wilcoxon rank test for post hoc testing was used for analyzing the continuous data. The chi-square or Fisher exact test was used for analyzing the categorical data. The Kruskal–Wallis test combined with the Wilcoxon rank test for post hoc testing was used for analyzing the ordinal data. For intragroup comparisons of continuous data, we used paired *t*-tests or Wilcoxon signed-rank tests.

For all statistical analyses, two-sided hypothesis tests were used, and the threshold of statistical significance of the two-tailed *P* value was 0.05. For the pairwise comparison between the three groups, the test level was adjusted to 0.0167 (0.05/3) according to the Bonferroni correction. The statistical analysis was performed using SAS V9.4 software (SAS Institute, Cary, NC, United States).

## 3. Results

There were 153 long-term follow-up patients, which comprised 141 patients who completed the 52-week follow-up of disease activity and 105 patients who completed the 52-week follow-up of radiological progression.

### 3.1. Patient Characteristics

Of the 153 patients followed up, 49 were in the TCM group, 49 were in the WM group, and 55 were in the IM group. In terms of sex, age, course of the disease, and other general population data, there were no significant differences between the three groups (*p* > 0.05; Table 1). In the baseline data, there was a higher proportion of women.

Patients were mainly middle-aged and older adults, which is consistent with the typical characteristics of RA patients. In terms of disease course, the data of the three groups were similar.

### 3.2. Clinical Efficacy

#### 3.2.1. Radiographic Outcome

Results showed that baseline TSS, JSN, and JE scores did not differ among the three groups. At 52 weeks, the TSS, JSN, and JE scores did not differ significantly between the three groups (Table 2).

**Figure 2:**
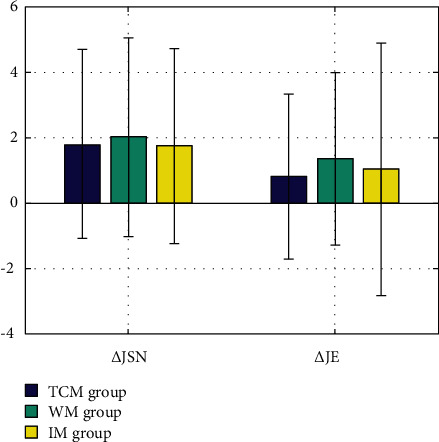
Comparison of JSN and JE among the three groups.

TSS, JSN, and JE of the three groups at baseline and 52 weeks were calculated separately. It was found that the radiology of the WM group has progressed relatively quickly, and that of the TCM group came next, and the change value of IM group was the smallest. But the difference among them was not statistically significant (Table 2 and Figure 2).

**Figure 3:**
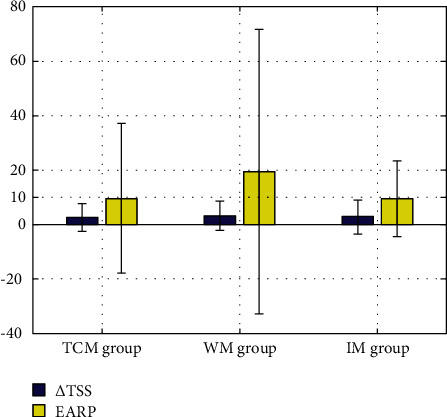
Comparison of TSS and EARP among the three groups.

Based on baseline TSS and disease course data, the estimated annual radiographic progression (EARP) was calculated, which is the TSS/disease course at baseline. There was no significant difference among the three groups or pairwise comparisons between groups (Table 2). Comparisons of the EARP with actual annual TSS changes of each group showed that the actual changes in each group were significantly lower than the baseline EARP, which suggested that the intervention measures were highly effective. Moreover, the radiological progression in three groups was well controlled (Figure 3).

A horizontal comparison was performed between the TCM, WM, and IM groups, and the change rates of TSS, JSN, and JE scores were calculated for each group. The average rate of progression at each evaluation point was calculated by (TSS − baseline score)/treatment time. Data showed that the three groups had similar rates of change of all three scores, as shown in Table 3. A horizontal comparison of the rates of change among the three groups indicated that they were comparable.

**Figure 4:**
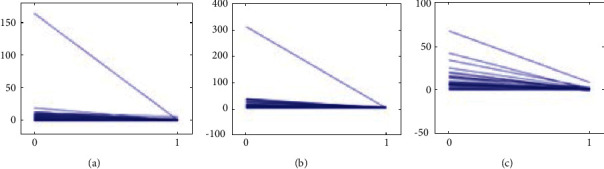
Comparison of TSS change among the three groups: (a) TCM groups, (b) WM group, and (c) IM group.

**Figure 5:**
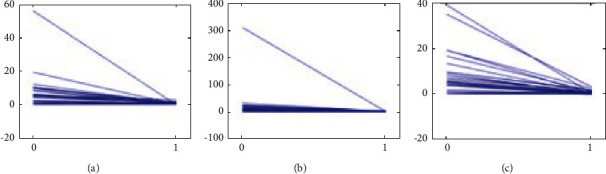
Comparison of JSN change among the three groups: (a) TCM groups, (b) WM group, and (c) IM group.

**Figure 6:**
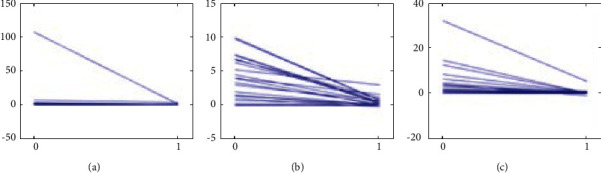
Comparison of JE change among the three groups: (a) TCM groups, (b) WM group, and (c) IM group.

TSS, JSN, and JE scores after treatment were compared longitudinally with the baseline change rate. The baseline change rate was calculated by baseline TSS/disease course. The change rate of the three groups after treatment was significantly slower than the baseline change rate before treatment. The three groups of treatments could decrease the radiographic progression of RA patients (Figures 4–6).

#### 3.2.2. Disease Activity Evaluation

After treatment, CRP, ESR, TJC, SJC, VAS, PhGA, PGA, and DAS28 in all three treatment groups decreased significantly from baseline to 52 weeks. There were no significant differences in the degree of improvement among the three groups, as shown in Tables 4 and 5.

### 3.3. Correlation Analysis between Radiology Score and DAS28

The correlation analysis showed that there was a significant correlation between the change values of the DAS28 and TSS. The change in DAS28 was smaller than that in TSS, as shown in Table 6. For those whose DAS28 change value was less than 1.41, the average TSS progression was 3.43. For those whose DAS28 change value was between 1.41 and 2.90 as well as above 2.90, the TSS change values were both 2.74.This association was observed between the DAS28 change value and both the JSN and baseline JE scores. This is consistent with previous findings.

### 3.4. Adverse Events

During the 24-week treatment and 52-week follow-up, there were no serious adverse events in any of the three groups, and none of the patients withdrew from the study because of the adverse reactions. The main adverse events were skin erythema, skin edema, skin itching, gastrointestinal reactions, blood system damage, and irregular menstruation. The incidence of adverse events in the TCM groups was the lowest at only 12 person-times, which included five cases of skin erythema, three cases of skin edema, two cases of skin pruritus, one case of gastrointestinal reactions, and one case of blood system damage. However, the incidence of adverse events in the WM and IM groups was relatively higher at 26 and 23 person-times, respectively (Table 7).

## 4. Discussion

RA is a chronic disabling autoimmune disease. With a prolonged disease course, the disability rate of patients increases significantly. Many experts now consider long-term management of disease activity and alleviation of related joint damage as the ultimate goals of RA management. This study focused on the clinical endpoint of RA bone destruction, which affects the long-term prognosis and life quality of RA patients. A follow-up clinical study was conducted to evaluate the long-term impact of TCM on the radiological progression of RA to promote the use of TCM.

Because RA is a chronic disease, we followed up a portion of patients in the study of “Rheumatoid arthritis TCM syndrome and comprehensive treatment plan for its 24-week RCT” at week 52 using similar evaluations. The current research was a long-term extension of the “24-week RCT” and is an innovative application based on real-world research concepts.

After the “24-week RCT” study, we adopted an observational comparative efficacy (CER) study design. As early as 2009, the American Agency for Healthcare Research and Quality (AHRQ) proposed the CER research method based on the concept of furthering detailed effectiveness research. CER is a highly valuable research method [14–16]. We noticed that a treatment plan that provided ideal outcomes in a well-controlled experimental environment, such as an RCT, performed poorly in clinical practice. In this study, at the end of the 24-week RCT, we used the observational CER research method from 24 to 52 weeks. The observational CER method provided a real-world portrayal and systematic evaluation of true long-term curative effects of a comprehensive QingreHuoxue treatment in RA patients. In the real world, the clinical value and significance of long-term follow-up of diseases are considerable and vital for determining the actual clinical efficacy of QRHXD/QRHXEP for RA. Long-term follow-up of RA patients is challenging; thus, the information obtained from this study is invaluable. Observational CER methods have offered new opportunities for clinical research and are an innovative application of research design methods based on real-world research concepts, which can provide valuable guidance for practical clinical work.

Moderate to severe inflammation is a significant contributor to RA bone destruction. In RA patients, local synovitis promotes the differentiation and proliferation of osteoclasts, destroys bone structure, erodes local bone, and affects the normal function of joints and bones. Only when local and systemic inflammations have been effectively controlled, can the occurrence of RA bone destruction be effectively reduced. MTX is currently the first-line basic anchoring drug in the treatment of RA. Based on the concept of early and standard treatments, if DMARD treatment is ineffective, a combined treatment plan can be used [17]. In this study, the classic combination of MTX and HCQ was selected for the EM treatment, which was compared head to head with the comprehensive TCM and IM treatment plans.

QRHXD/QRHXEP can reduce RA disease activity and is extremely safe. After completion of the multicenter RCT of the comprehensive TCM program for clearing heat and promoting blood circulation, we continued to follow up the patients and monitor disease activity under realistic conditions. The 52-week follow-up results showed that all three groups had similar levels of disease activity. Although there was no significant difference, there was a higher rate of compliance with DAS28 in the IM group.

The QRHXD/QRHXEP was effective in delaying the progression of RA bone destruction. The radiological progression and joint function of RA patients are key factors that affect their long-term prognosis and quality of life. This study indicated that TCM had a positive effect on inhibiting the bone destruction in RA patients and was no less inferior to the classic Western medicine treatment. In addition, we observed that the IM group had relatively lower scores of TSS, JSN, and JE than those of TCM group and WM group. However, there was no significant difference among the three groups, which may be due to the relatively small number of cases. We plan to conduct large-scale clinical studies in the future to further optimize the treatment plans for RA.

A recent meta-analysis [18] showed that TCM significantly improved the bone density, reduced the level of serum matrix metalloproteinase 3, and protected the bone condition of RA patients. At present, there are an increasing number of clinical treatments that intervene in RA bone destruction. Yet, there have been relatively few long-term efficacy studies. Additional randomized controlled trials with objective study designs are needed to obtain rich high-level clinical evidence to support TCM intervention for RA bone destruction and ultimately benefit more RA patients.

We found that there were no serious adverse events during the entire study. Furthermore, we found that the TCM group had the lowest incidence of adverse events and the highest safety among the three groups.

This study has several limitations. Firstly, the inherent limitations of study includes potential bias of open-label design and populations possibly representing only responders to therapy. However, properly designed and conducted open-label extension studies can provide rigorous information on long term efficacy of new therapy. Secondly, because of funding constraints, the radiographic images included in this study were only radiographic images of the hands and wrists and did not include those of the feet. Studies have shown that the joints of the feet are usually affected more easily and earlier than the joints of the hands. Therefore, including data on feet may help improve the sensitivity of assessment in the joint damage of early RA [19–22]. We plan to conduct further clinical trials in the future to evaluate the effect of TCM on RA bone destruction.

## 5. Conclusion

We used a comprehensive QingreHuoxue treatment to treat active RA and conducted a long-term follow-up of patients. This was a real-world observational comparative study that evaluated the long-term effects of TCM treatment on RA. We found that a comprehensive QingreHuoxue treatment delayed the radiological progression of RA and continued to reduce disease activity. The following conclusions are drawn:

1. The program of clearing heat and promoting blood circulation had a therapeutic effect on RA bone destruction. The TCM group significantly slowed radiological progression and was not inferior to the combined MTX and HCQ treatment group.
2. TCM treatment helped RA patients achieve rapid relief of symptoms (such as joint swelling and pain), reduce inflammatory indicators (such as ESR), and reduce disease activity.
3. The incidence of adverse events was the lowest, and safety was the highest in RA patients who were offered the TCM treatment.

## Figures and Tables

**Table 1:** Comparison of the baseline population data among the three groups.

Items		TCM group	WM group	IM group	*P*-value
		(*n* = 49)	(*n* = 49)	(*n* = 55)	
Gender	Male, *N* (%)	4 (8.16)	10 (20.41)	9 (16.36)	0.2237
	Female, *N* (%)	45 (91.84)	39 (79.59)	46 (83.64)	
Age	Mean (SD)	47.59 (11.11)	46.31 (10.71)	49.40 (10.07)	0.2998
Course	Mean (SD)	28.27 (37.61)	32.42 (37.08)	21.05 (16.62)	0.1123

**Table 2:** Comparison of TSS among the three groups.

	TCM group, *N* = 33	WM group, *N* = 35	IM group, *N* = 37	*P*-value
*TSS baseline*				
Mean (SD)	7.48 (8.54)	19.23 (24.07)	17.62 (29.53)	0.3078
Median (Q1, Q3)	6 (1, 9)	10 (2, 33)	6 (0, 17)	

*JSN baseline*				
Mean (SD)	5.42 (4.66)	14.40 (18.03)	11.22 (16.44)	0.2763
Median (Q1, Q3)	5 (0, 8)	7 (1, 21)	5 (0, 16)	

*JE baseline*				
Mean (SD)	2.06 (5.23)	4.80 (6.57)	6.41 (13.94)	0.135
Median (Q1, Q3)	0 (0, 2)	1 (0, 9)	1 (0, 3)	

*EARP*				
Mean (SD)	9.61 (27.45)	19.45 (52.29)	9.56 (13.84)	0.3379
Median (Q1, Q3)	4 (0.35, 9.60)	6 (1, 24)	5.54 (0, 11.33)	

*TSS (52 weeks)*				
Mean (SD)	10.12 (11.77)	22.57 (26.99)	20.43 (34.86)	0.3274
Median (Q1, Q3)	7 (2, 14)	12 (3, 34)	6 (1, 17)	

*JSN (52 weeks)*				
Mean (SD)	7.24 (6.30)	16.43 (19.02)	12.97 (19.03)	0.3567
Median (Q1, Q3)	6 (2, 11)	8 (2, 23)	6 (1, 16)	

*JE (52 weeks)*				
Mean (SD)	2.88 (6.96)	6.17 (8.53)	7.46 (16.83)	0.2924
Median (Q1, Q3)	0 (0, 2)	1 (0, 12)	1 (0, 6)	

*△TSS*				
Mean (SD)	2.64 (5.02)	3.34 (5.31)	2.81 (6.18)	0.4997
Median (Q1, Q3)	1 (0, 3)	1 (0, 4)	1 (0, 4)	

*△JSN*				
Mean (SD)	1.82 (2.88)	2.03 (3.04)	1.76 (2.99)	0.8145
Median (Q1, Q3)	1 (0, 2)	1 (0, 3)	1 (0, 2)	

*△JE*				
Mean (SD)	0.82 (2.54)	1.37 (2.64)	1.05 (3.86)	0.1684
Median (Q1, Q3)	0 (0, 0)	0 (0, 2)	0 (0, 0)	

Note: TSS is the total Sharp score; JSN is the joint gap narrow score; JE is the joint erosion score; the EARP is baseline △TSS/disease duration; △TSS is 52-week TSS − baseline TSS; and △JE is 52-week JE − baseline JE.

**Table 3:** Change of TSS, JSN, and JE scores in the three groups.

	TCM group, *N* = 33	WM group, *N* = 35	IM group, *N* = 37	*P*-value
*The rate of change in TSS*				
Mean (SD)	0.31 (0.53)	0.34 (0.78)	0.21 (0.66)	0.2997
Median (Q1, Q3)	0.08 (0.00, 0.33)	0.08 (0.00, 0.30)	0.00 (0.00, 0.20)	

*The rate of change in JSN*				
Mean (SD)	0.25 (0.52)	0.35 (0.80)	0.10 (0.20)	0.447
Median (Q1, Q3)	0.00 (0.00, 0.27)	0.04 (0.00, 0.25)	0.00 (0.00, 0.13)	

*The rate of change in JE*				
Mean (SD)	0.07 (0.21)	0.13 (0.23)	0.17 (0.44)	0.1472
Median (Q1, Q3)	0.00 (0.00, 0.00)	0.00 (0.00, 0.15)	0.00 (0.00, 0.00)	

Note. Baseline rate = baseline TSS/course. Average rate of progression at each evaluation point of time = (TSS − baseline score)/treatment time.

**Table 4:** Disease activity comparison (ITT) between baseline and week 52.

Items	Baseline			52 weeks			*P*-value
	TCM group, *N* = 36	WM group, *N* = 35	IM group, *N* = 41	TCM group, *N* = 36	WM group, *N* = 35	IM group, *N* = 41	
*ESR (mm/h)*							
Mean (SD)	47.65	35.9	41.62	33.74	21.45	23.04	0.0843
	(29.36)	(21.72)	(27.42)	(28.19)	(18.67)	(16.08)	
Median (Q1, Q3)	34	31	33	23.5	14	20	
	(23, 68)	(23, 46)	(21, 65)	(13, 51)	(9, 29)	(10, 30)	

*VAS (mm)*							
Mean (SD)	54.49	54.24	54.85	19.73	21.3	18.38	0.7379
	(16.65)	(16.13)	(17.74)	(19.16)	(18.18)	(16.26)	
Median (Q1, Q3)	52	50	53	20	20	11	
	(40, 60)	(45, 60)	(40, 70)	(10, 30)	(10, 30)	(5.50, 30)	

*PGA (mm)*							
Mean (SD)	58.33	55.49	60.15	23.78	24.02	20.87	0.7631
	(17.55)	(19.14)	(19.45)	(17.74)	(20.26)	(15.24)	
Median (Q1, Q3)	60	50	60	20	15	20	
	(50, 70)	(45, 70)	(50, 80)	(10, 30)	(10, 40)	(10, 30)	

*PhGA (mm)*							
Mean (SD)	57	53.86	54.62	26.42	26.45	21.5	0.3392
	(14.67)	(15.06)	(18.87)	(17.89)	(18.9)	(14.71)	
Median (Q1, Q3)	60	50	60	30	20	20	
	(50, 70)	(45, 65)	(40, 70)	(10, 30)	(10, 40)	(10, 30)	

*TJC*							
Mean (SD)	10.78	9.96	10.36	3.53	3.91	2.63	0.3329
	(6.73)	(5.57)	(7.42)	(4.57)	(4.49)	(2.64)	
Median (Q1, Q3)	9	10	8	2	3	2	
	(6, 15)	(5, 13)	(5, 14)	(1, 5)	(1.50, 4)	(0, 4)	

*SJC*							
Mean (SD)	7.92	7.71	7.62	1.89	1.66	1.12	0.4277
	(5.23)	(5.32)	(5.31)	(2.41)	(2.89)	(1.62)	
Median (Q1, Q3)	7	6	6	1	0	1	
	(4, 11)	(3, 11)	(4, 10)	(0, 3)	(0, 2.50)	(0, 1)	

*DAS28*							
Mean (SD)	5.84	5.57	5.62	3.61	3.34	3.2	0.4752
	(0.98)	(0.99)	(1.26)	(1.45)	(1.33)	(1.05)	
Median (Q1, Q3)	5.9	5.44	5.47	3.14	3.17	2.97	
	(5.17, 6.36)	(4.93, 6.32)	(4.68, 6.69)	(2.75, 4.30)	(2.61, 4.01)	(2.44, 3.98)	

**Table 5:** Disease activity comparison (PP) between baseline and week 52.

Items	Baseline			52 weeks			*P*-value
	TCM group *N* = 36	WM group *N* = 35	IM group *N* = 41	TCM group *N* = 36	WM group *N* = 35	IM group *N* = 41	
*ESR (mm/h)*							
Mean (SD)	46.94	32.74	43.90	33.67 (28.38)	19.09 (15.92)	24.45 (15.65)	0.0563
	(28.71)	(19.51)	(28.67)				
Median (Q1, Q3)	34.00	28.00	38.00	23.00	14.00	22.50	
	(22.5, 66.5)	(20.0, 44.0)	(25.0, 69.0)	(13.0, 51.0)	(9.0, 25.0)	(10.0, 31.0)	

*VAS (mm)*							
Mean (SD)	54.75	52.37	53.90	18.56	19.34	19.54	0.9146
	(16.55)	(16.26)	(16.68)	(17.64)	(15.96)	(16.98)	
Median (Q1, Q3)	53.50	50.00	50.00	15.00	15.00	20.00	
	(40.0, 62.5)	(40.0, 60.0)	(40.0, 65.0)	(7.5, 30.0)	(10.0, 30.0)	(10.0, 30.0)	

*PGA (mm)*							
Mean (SD)	59.11	54.97	61.41	22.92	24.77	23.05	0.985
	(17.06)	(19.80)	(18.97)	(16.61)	(20.77)	(15.29)	
Median (Q1, Q3)	60.00	50.00	60.00	20.00	20.00	20.00	
	(50.0, 70.0)	(40.0, 70.0)	(50.0, 80.0)	(10.0, 30.0)	(10.0, 40.0)	(10.0, 30.0)	

*PhGA (mm)*							
Mean (SD)	57.03	53.40	53.61	25.25	24.40	22.27	0.7998
	(14.60)	(15.87)	(17.48)	(17.32)	(18.01)	(15.14)	
Median (Q1, Q3)	60.00	50.00	50.00	22.50	20.00	20.00	
	(50.0, 70.0)	(40.0, 70.0)	(40.0, 70.0)	(10.0, 30.0)	(10.0, 40.0)	(10.0, 30.0)	

*TJC*							
Mean (SD)	11.03	11.00	9.51	1.69	1.51	0.95	0.3336
	(7.30)	(5.78)	(7.66)	(2.12)	(2.20)	(1.53)	
Median (Q1, Q3)	9.00	11.00	7.00	1.00	0.00	0.00	
	(5.0, 15.5)	(6.0, 14.0)	(4.0, 13.0)	(0.0, 2.5)	(0.0, 3.0)	(0.0, 1.0)	

*SJC*							
Mean (SD)	8.44	7.69	7.20	3.89	3.54	2.44	0.3183
	(5.66)	(5.35)	(5.19)	(4.89)	(3.53)	(2.41)	
Median (Q1, Q3)	8.00	6.00	5.00	2.00	3.00	2.00	
	(3.5, 11.5)	(3.0, 11.0)	(4.0, 8.0)	(0.5, 5.0)	(2.0, 4.0)	(0.0, 4.0)	

*DAS28*							
Mean (SD)	5.87	5.56	5.52	3.62	3.21	3.22	0.2902
	(1.00)	(1.06)	(1.22)	(1.51)	(1.19)	(0.97)	
Median (Q1, Q3)	5.90	5.50	5.38	3.15	3.20	3.15	
	(5.16, 6.49)	(4.80, 6.31)	(4.59, 6.60)	(2.75, 4.79)	(2.31, 4.01)	(2.65, 3.98)	

**Table 6:** Correlation between DAS28 change values and TSS changes.

		△DAS28		
		<1.41 (*n* = 30)	1.41–2.90 (*n* = 50)	>2.90 (*n* = 23)
△TSS	Mean (SD)	3.43 (6.49)	2.74 (4.65)	2.74 (5.88)
	Median (Q1, Q3)	1.5 (0, 4.75)	1 (0, 4)	0 (0, 2.5)
△JSN	Mean (SD)	2.07 (2.76)	1.86 (2.86)	1.74 (3.38)
	Median (Q1, Q3)	1 (0, 3.75)	0.5 (0, 2.75)	0 (0, 1)
△JE	Mean (SD)	1.43 (4.22)	0.88 (2.31)	1.09 (2.75)
	Median (Q1, Q3)	0 (0, 0.75)	0 (0, 1)	0 (0, 0.5)

Note. △TSS was 52-week TSS − baseline TSS; △JSN was 52-week JSN − baseline JSN; △JE was 52-week JE − baseline JE. Comparison between the three groups at *p* < 0.05.

**Table 7:** Adverse events in three groups.

	TCM group	WM group	IM group
Total	12	26	23
Erythema on the skin	5	5	0
Skin edema	3	3	1
Itchy skin	2	5	5
Gastrointestinal reaction	1	6	8
Damage to the blood system	1	0	0
Menstruation is not normal	0	1	0
Stomachache	0	1	0
Headache, dizziness	0	2	2
Hair loss	0	2	2
Proteinuria increases	0	1	1
Upper respiratory tract infection	0	0	1
Urinary tract infection	0	0	1
Arrhythmia	0	0	1
Insomnia	0	0	1

## Data Availability

The original contributions presented in the study are included in the article/Supplementary Material; further inquiries can be directed to the corresponding authors.

## Supplementary Materials

Supplementary Materials File 1: supplementary materials_data_baseline. The supplementary materials are the original data of this study, mainly including the basic information of patients, grouping, disease activity, and X-ray Sharp score. The basic information of the subjects includes the name, gender, year of birth, and course of disease. Disease activity was recorded by the researchers at baseline, including DAS28 (28-joint count Disease Activity Score), CRP (C-reactive protein), ESR (erythrocyte sedimentation rate), TJC (tender joint count), SJC (swollen joint count), VAS (visual analogue scale), PhGA (physician's global assessment of disease activity), PGA (patient's global assessment of disease activity), and HAQ (Health Assessment Questionnaire). Subjects underwent radiological progression analysis at baseline, which involved frontal X-rays of both hands and wrists. Two radiologists read and analyzed the radiographic images according to the Sharp scoring system revised by van der Heijde. The radiologists had no knowledge of the treatment allocation, the chronology of radiographs, or patients' clinical responses. The Sharp scoring system is revised by van der Heijde, including TSS (total Sharp score), JSN (joint gap narrow score), and JE (joint erosion score). The sum of joint erosion (JE) score and joint space narrowing (JSN) was the value of total Sharp score (TSS). File 2: supplementary materials_data_52w. The supplementary materials are the original data of this study, mainly including the basic information of patients, grouping, disease activity and X-ray sharp score. The basic information of the subjects includes the name, gender, and year of birth. Disease activity was recorded by the researchers at 52 weeks of follow-up, including DAS28 (28-joint count Disease Activity Score), CRP (C-reactive protein), ESR (erythrocyte sedimentation rate), TJC (tender joint count), SJC (swollen joint count), VAS (visual analogue scale), PhGA (physician's global assessment of disease activity), PGA (patient's global assessment of disease activity), and HAQ (Health Assessment Questionnaire). Subjects underwent radiological progression analysis at week 52, which involved frontal X-rays of both hands and wrists. Two radiologists read and analyzed the radiographic images according to the Sharp scoring system revised by van der Heijde. The radiologists had no knowledge of the treatment allocation, the chronology of radiographs, or patients' clinical responses. The Sharp scoring system is revised by van der Heijde, including TSS (total Sharp score), JSN (joint gap narrow score), and JE (joint erosion score). The sum of joint erosion (JE) score and joint space narrowing (JSN) was the value of total Sharp score (TSS) .
