# Supplementary material for: The Impact of Traditional Chinese Medicine QingreHuoxue Treatment and the Combination of Methotrexate and Hydroxychloroquine on the Radiological Progression of Active Rheumatoid Arthritis: A 52-Week Follow-Up of a Randomized Controlled Clinical Study
Source: Evid Based Complement Alternat Med. 2022 Apr 12;2022:5808400. doi: 10.1155/2022/5808400 (PMC9019417; doi:10.1155/2022/5808400)
Supplement: Supplementary Materials — File 1: supplementary materials_data_baseline. The supplementary materials are the original data of this study, mainly including the basic information of patients, grouping, disease activity, and X-ray Sharp score. The basic information of the subjects includes the name, gender, year of birth, and course of disease. Disease activity was recorded by the researchers at baseline, including DAS28 (28-joint count Disease Activity Score), CRP (C-reactive protein), ESR (erythrocyte sedimentation rate), TJC (tender joint count), SJC (swollen joint count), VAS (visual analogue scale), PhGA (physician's global assessment of disease activity), PGA (patient's global assessment of disease activity), and HAQ (Health Assessment Questionnaire). Subjects underwent radiological progression analysis at baseline, which involved frontal X-rays of both hands and wrists. Two radiologists read and analyzed the radiographic images according to the Sharp scoring system revised by van der Heijde. The radiologists had no knowledge of the treatment allocation, the chronology of radiographs, or patients' clinical responses. The Sharp scoring system is revised by van der Heijde, including TSS (total Sharp score), JSN (joint gap narrow score), and JE (joint erosion score). The sum of joint erosion (JE) score and joint space narrowing (JSN) was the value of total Sharp score (TSS). File 2: supplementary materials_data_52w. The supplementary materials are the original data of this study, mainly including the basic information of patients, grouping, disease activity and X-ray sharp score. The basic information of the subjects includes the name, gender, and year of birth. Disease activity was recorded by the researchers at 52 weeks of follow-up, including DAS28 (28-joint count Disease Activity Score), CRP (C-reactive protein), ESR (erythrocyte sedimentation rate), TJC (tender joint count), SJC (swollen joint count), VAS (visual analogue scale), PhGA (physician's global assessment of disease activity), [file 5808400.f1.zip › 5808400.f1/supplementary materials_data_52w.pdf]

original source data(52w)

Group	name	gender (1=male, 2=female)	year of birth	24w-52w_treat (non=0, treatment=1)	24w-52w_med (TCM=0, WM=1, IM=2, non=3)	TJC (52w)	SJC (52w)	VAS (52w)	PhGA(52w)	PGA(52w)	HAQ(52w)	ESR (52w)	DAS28(52w)	TSS(52w)	JSN(52w)	JE(52w)
2	Hyi C	2	1955	1	2	3	0	10	10	10	2	17	3.09	25	16	9
2	Yru C	2	1959	0		3	2	0	50	40	5	30	3.74	18	16	2
1	Fxi C	2	1969	0		14	4	30	30	30	20	70	6.04	3	2	1
1	Hgu C	1	1968	0		11	1	40	30	40	8	25	4.95	2	2	0
2	Sh Ch	2	1988	0		11	1	0	10	5	0	3	2.89	0	0	0
3	Sho C	1	1964	0		2	0	20	10	10	0	38	3.62	0	0	0
1	Zli C	2	1981	1	2	3	3	40	40	40	5	4	2.99	22	17	5
1	Gxi D	2	1964	1	2	9	9	80	70	80	17	96	6.85	6	4	2
1	Yli D	2	1973	0		4	0	20	30	20	1	11	3.07	6	6	0
1	Lxi D	2	1952	0		4	0	10	10	20	2	13	3.05	21	15	6
3	We Fa	1	1967	1	1	1	1	5	5	10	0	7	2.27	77	54	23
2	Eyu F	1	1951	0		1	0	15	15	10	1	9	2.31	13	13	0
3	Pi Fe	2	1950	0		1	1	10	40	40	1	73	3.98	3	3	0
1	Lli G	2	1970	0		5	2	30	30	30	3	90	5.22	8	6	2
3	Xyu G	2	1955	0		0	0	0	5	7	0	8	1.46	23	17	6
1	Mxi G	2	1955	0		1	0	0	20	25	0	44	3.2	16	15	1
3	Jxi G	2	1960	0		3	0	20	20	20	0	8	2.7	2	2	0
2	Rfe G	2	1957	0		5	2	50	40	50	3	13	4.15	69	52	17
2	Wqi G	2	1968	1	2	1	1	20	20	10	0	9	2.66	1	1	0
3	Jyi H	2	1977	1	1	2	1	10	10	10	0	12	2.95	17	16	1
3	Qru H	2	1956	0		0	0	0	40	20	9	51	2.75	106	64	42
3	We Ha	2	1965	0		2	1	30	30	30	7	33	3.94	15	14	1
2	Jbo H	2	1982	0		0	0	0	0	0	0	3	0.77	34	23	11
3	Jxi J	2	1964	0		2	2	6	16	18	0	10	2.88	0	0	0
1	Lli J	2	1980	0		1	1	3	3	4	0	5	2.01	23	19	4

Group	name	gender（1=male, 2=female ）	year of birth	24w-52w_treat（non=0，treatment=1）	24w-52w_med（TCM=0，WM=1，IM=2，non=3）	TJC（52w）	SJC（52w）	VAS（52w)	PhGA(52w)	PGA(52w)	HAQ(52w)	ESR（52w）	DAS28(52w)	TSS(52w)	JSN(52w)	JE(52w)
1	Lu Ji	2	1979	0		1	1	10	10	10	0	27	3.29	43	13	30
1	Xqi K	2	1954	0		0	0	0	30	10	0	58	2.84	0	0	0
2	Xqi K	2	1971	0		2	3	50	40	70	9	18	4.01	44	30	14
1	Csh L	2	1962	0		10	4	20	60	40	12	40	5.19	2	2	0
1	Jho L	2	1959	0		1	0	20	10	20	0	16	2.78	9	7	2
3	Jyu L	2	1996	0		3	3	60	10	20	2	26	4.59	171	83	88
1	Jpi L	2	1951	1	3	0	0	20	30	20	0	58	3.13	2	2	0
1	Sju L	2	1981	0		6	7	0	50	50	3	61	4.99	5	5	0
3	Xbo L	1	1979	0		4	0	30	30	30	0	19	3.6	3	2	1
2	Xhu L	2	1972	0		2	0	10	10	10	2	13	2.72	13	13	0
3	Fya L	2	1959	0		4	3	10	10	10	3	27	4.05	0	0	0
3	Ghu L	2	1955	0		8	6	30	30	30	1	50	5.43	13	10	3
3	Hti L	2	1953	1	1	0	0	0	10	0	0	9	1.54	0	0	0
2	Jla L	2	1969	0		0	0	10	50	70	0	14	1.99	5	5	0
1	Jhu L	2	1977	0		0	0	0	0	0	0	22	2.16	0	0	0
3	Li Li	2	1969	0		5	0	40	40	40	3			5	4	1
3	Mi Li	2	1955	0		6	6	10	20	20	5	47	4.89	48	31	17
2	Syi L	2	1958	0		0	0	0	3	5	0	7	1.36	2	2	0
2	Xme L	2	1964	1	2	23	15	80	80	60	1	74	7.91	77	54	23
2	Wji L	2	1983	0		0	2	10	10	10	0	8	2	87	68	19
2	Bju M	2	1977	0		4	0	30	40	30	2	31	3.94	13	13	0
1	Gpi M	2	1965	1	1	1	7	0	50	10	1	24	3.53	11	11	0
2	Wyi M	2	1988	0		1	1	47	51	57	3	49	4.23	68	47	21
2	Ji Me	2	1964	0		2	0	10	10	10	0	18	2.95	15	14	1
1	Yhu M	2	1974	0		0	0	0	5	1	0	5	1.13	5	5	0
1	Xro N	2	1978	0		21	7	80	80	80	20	117	7.76	9	7	2

Group	name	gender （1=male, 2=female ）	year of birth	24w-52w_treat （non=0, treatment=1 ）	24w-52w_med （TCM=0, WM=1, IM=2, non=3 ）	TJC （52w ）	SJC （52w ）	VAS （52w)	PhGA(52w)	PGA(52w)	HAQ(52w)	ESR （52w ）	DAS28(52w)	TSS(52w)	JSN(52w)	JE(52w)
3	Yli P	2	1962	1	2	7	3	25	30	10	3	58	5.16	3	2	1
3	Jju R	2	1972	0		4	0	20	20	30	0	6	2.65	56	38	18
3	Yli S	1	1954	0		2	1	60	60	60	8	52	4.69	10	10	0
3	Cxi S	2	1972	0		3	0	30	30	30	1	19	3.45	0	0	0
1	Syu S	2	1953	0		0	0	0	10	0	0	51	2.75	4	3	1
2	Xla S	2	1955	0		18	7	40	40	40	12	20	5.77	4	1	3
3	Mpi T	2	1956	0		0	0	0	0	0	0	29	2.36	54	18	36
3	Ji Ti	2	1958	1	1	11	6	30	30	30	6	45	5.63	11	5	6
2	Xya T	2	1976	0		3	3	30	30	30	9	13	3.67	77	54	23
2	Sde W	1	1968	1	2	1	0	0	40	0	0	63	3.46	62	35	27
2	Hqi W	2	1963	0		4	0	40	40	40	1	39	4.24	2	2	0
2	Jyu W	1	1953	0		4	0	10	40	10	0	1	1.25	34	22	12
2	Li Wa	2	1982	0		4	0	10	10	10	1	14	3.1	6	6	0
1	Lju W	2	1975	0		7	4	20	25	20	2	34	4.79	16	16	0
1	Mi Wa	2	1982	0		5	3	40	30	10	6	12	4.04	7	6	1
3	Pi Wa	1	1959	0		0	0	0	0	0	0	22	2.16	0	0	0
3	Rha W	1	1963	1	1	4	1	10	10	10	1	4	2.51	1	1	0
2	Sli W	2	1957	0		3	0	30	30	30	0	36	3.9	2	2	0
2	Sfe W	2	1954	0		5	7	10	10	10	4	11	3.81	4	1	3
1	Szh W	2	1962	0		2	2	5	11	10	0	13	3.05	14	9	5
3	Xxi W	2	1958	1	1	5	1	20	40	20	4	10	3.42	1	0	1
1	Xho W	2	1970	0		1	1	10	10	5	0	9	2.52	0	0	0
1	Xyi W	2	1955	0		0	0	10	10	10	0	15	2.04	5	4	1
2	Yju W	1	1973	0		4	0	10	10	10	0	7	2.61	0	0	0
2	Cli W	1	1981	0		6	8	0	80	75	23	75	5.18	7	7	0
3	Sju W	2	1959	0		2	0	10	10	10	0	35	3.42	10	8	2

Group	name	gender（1=male, 2=female ）	year of birth	24w-52w_treat（non=0, treatment=1 ）	24w-52w_med（TCM=0, WM=1, IM=2, non=3 ）	TJC（52w ）	SJC（52w ）	VAS（52w)	PhGA(52w)	PGA(52w)	HAQ(52w)	ESR（52w ）	DAS28(52w)	TSS(52w)	JSN(52w)	JE(52w)
2	We We	2	1953	0		3	3	30	20	10	4	29	4.24	12	11	1
3	Gue W	2	1959	0		1	0	10	10	10	0	23	2.89	6	6	0
2	Yme W	2	1982	1	2	2	0	50	50	10	0	6	2.75	0	0	0
2	Xme W	2	1965	1	1	0	1	10	20	10	0	21	2.56	3	0	3
1	Ypi X	2	1969	0		4	0	20	20	20	0	11	3.07	0	0	0
1	Hya X	2	1977	0		2	6	30	40	30	7	25	4.16	9	9	0
3	Swe X	2	1983	0		2	0	10	10	10	0	8	2.38	2	2	0
2	Hni Y	1	1972	0		2	0	40	20	40	2	14	3.2	10	8	2
1	Ju Ya	2	1949	0		0	0	0	50	30	1	5	1.13	8	8	0
2	Li Ya	2	1985	0		3	0	10	10	10	0	25	3.36	5	5	0
3	Lxi Y	2	1970	0		0	0	2	5	3	6	6	1.28	12	10	2
3	Xyu Y	2	1973	0		1	1	50	0	50	0	5	2.68	0	0	0
2	Xne Y	2	1966	0		3	0	20	20	20	0	3	2.01	6	5	1
3	Hxi Z	2	1956	0		2	1	0	50	50	9	29	3.43	17	10	7
3	Chu Z	2	1968	1	1	3	1	40	30	40	6	10	3.43	5	5	0
1	Fyi Z	2	1966	0		5	5	20	20	30	3	55	4.97	8	8	0
3	Jhu Z	2	1970	0		0	0	26	32	32	0	9	1.91	16	12	4
3	Jqi Z	2	1960	0		10	0	12	10	10	2	23	4.12	5	4	1
1	Qni Z	2	1979	0		5	0	20	20	20	0	36	4.03	0	0	0
1	Xya Z	2	1982	0		0	0	10	20	20	1	16	2.08	51	23	28
2	Xzh Z	2	1958	1	1	9	0	20	20	20	10	14	3.8	58	40	18
2	Zch Z	1	1963	0		9	0	20	20	20	0	NA		13	8	5
2	Mju Z	2	1984	0		3	0	30	20	30	2	12	3.13	0	0	0
3	Zyi Z	2	1987	0		3	0	10	10	10	3	24	3.33	23	20	3
3	Cne Z	2	1960	0		7	0	40	40	40	10	42	4.65	0	0	0
1	Mfa Z	2	1952	0		15	2	60	50	50	9	73	6.41	0	0	0

Group	name	gender（1=male, 2=female ）	year of birth	24w-52w_treat（non=0, treatment=1）	24w-52w_med（TCM=0, WM=1, IM=2, non=3）	TJC（52w）	SJC（52w）	VAS（52w）	PhGA(52w)	PGA(52w)	HAQ(52w)	ESR（52w）	DAS28(52w)	TSS(52w)	JSN(52w)	JE(52w)
3	Mi Zh	2	1969	0		5	1	30	30	30	1	20	4.05	34	26	8
1	Jme Z	2	1965	1	1	1	1	30	30	30	0	9	2.8	18	14	4
2	Rqi H	2	1971	quit												
3	Xyu W	2	1967	0		1	1	20	25	25	2	23	3.32			
3	Ju Wa	2	1978	0		1	1	10	10	10	0	8	2.44			
1	Sju Z	2	1957	0		1	1	25	30	30	1	84	4.3			
2	Lli H	2	1981	0		4	4	20	20	20	0	48	4.67			
1	Yi Wa	2	1967	1	0	1	0	10	10	10	1	16	2.64			
3	Ypi M	2	1963	0		0	1	30	30	35	1	17	2.69			
3	Hyu X	2	1957	quit												
3	Yme Z	2	1979	1	2	0	0	5	5	5	0	11	1.75			
2	Fro G	2	1952	0		7	3	40	40	40	5	13	4.32			
1	Sxi R	2	1966	quit												
3	Xi Ya	2	1968	0		0	0	0	30	30	0	30	2.38			
2	Xla Q	2	1963	quit												
1	Jli C	2	1964	0		2	4	30	35	30	9	NA				
2	Jji Z	2	1971	0		4	3	30	30	30	1	14	3.88			
2	Xwa L	2	1974	0		0	0	0	0	0	0	18	2.02			
3	Lju M	2	1954	quit												
2	Zyu L	1	1973	1	2	2	0	30	30	30	6	14	3.06			
1	Ju Wa	2	1967	quit												
1	Cfa Z	1	1949	0		0	0	10	15	10	0	NA				
3	Wxi Y	1	1964	0		3	0	50	50	50	11	17	3.65			
1	Ywe L	2	1983	1	1	2	4	30	30	30	1	20	3.88			
3	Szh Y	2	1951	0		5	3	25	35	35	8	19	4.15			
1	Lpi L	1	1953	1	2	0	0	0	5	10	0	49	2.72			

Group	name	gender（1=male, 2=female ）	year of birth	24w-52w_treat（non=0, treatment=1）	24w-52w_med（TCM=0, WM=1, IM=2, non=3）	TJC（52w）	SJC（52w）	VAS（52w）	PhGA(52w)	PGA(52w)	HAQ(52w)	ESR（52w）	DAS28(52w)	TSS(52w)	JSN(52w)	JE(52w)
2	Zlo Y	1	1964	quit												
2	Xta P	2	1985	0		2	2	20	20	10	0	NA				
1	Dzh Y	2	1975	0		1	0	35	35	40	0	15	2.95			
1	Lru Z	2	1962	quit												
3	Wdo L	1	1971	0		3	3	10	10	10	0	5	2.72			
2	Li Ma	2	1964	0		0	0	5	5	5	0	6	1.33			
1	Gya L	1	1990	0		5	2	10	10	10	1	7	3.15			
3	Hzh Z	1	1983	1	2	4	4	10	30	5	0	23	4.01			
1	Dfa Y	2	1950	1	2	2	0	10	15	15	3	30	3.31			
1	Yzh B	2	1962	quit												
3	Zpi S	2	1963	0		0	0	0	5	5	0	16	1.94			
1	Cta D	2	1986	0		4	2	30	30	30	4	23	4.13			
2	Yqi Z	2	1956	quit												
1	Mxi X	2	1979	0		0	0	10	10	10	0	23	2.34			
3	Cla A	2	1961	0		4	3	40	30	30	5	NA				
2	Ju Wa	2	1966	quit												
3	Dda D	2	1984	1	2	0	1	0	5	0	0	11	1.96			
3	Fme L	2	1971	0		0	0	20	30	25	4	31	2.69			
1	Jhu C	2	1965	0		2	2	10	30	30	4	NA				
3	Ji Zh	2	1970	quit												
2	Qso L	1	1975	1	1	7	3	40	40	40	1	53	5.31			
3	Fqi W	2	1976	0		0	0	20	30	20	4	NA				
3	Lfe W	2	1956	0		1	1	0	10	0	5	21	2.97			
2	Xme L	2	1975	0		2	2	0	10	10	0	16	3.13			

Note：DAS28 28-joint count Disease Activity Score,CRP C-reactive protein,ESR erythrocyte sedimentation rate,TJC tender joint count, SJC swollen joint count,VAS visual analogue scale,PhGA Physician’ s global assessment of disease activity,PGA Patient’ s global assessment of disease activity,HAQ Health Assessment Questionnaire,TSS total Sharp score,JSN joint gap narrow score,JE joint erosion score.
