# Supplementary material for: The Impact of Traditional Chinese Medicine QingreHuoxue Treatment and the Combination of Methotrexate and Hydroxychloroquine on the Radiological Progression of Active Rheumatoid Arthritis: A 52-Week Follow-Up of a Randomized Controlled Clinical Study
Source: Evid Based Complement Alternat Med. 2022 Apr 12;2022:5808400. doi: 10.1155/2022/5808400 (PMC9019417; doi:10.1155/2022/5808400)
Supplement: Supplementary Materials — File 1: supplementary materials_data_baseline. The supplementary materials are the original data of this study, mainly including the basic information of patients, grouping, disease activity, and X-ray Sharp score. The basic information of the subjects includes the name, gender, year of birth, and course of disease. Disease activity was recorded by the researchers at baseline, including DAS28 (28-joint count Disease Activity Score), CRP (C-reactive protein), ESR (erythrocyte sedimentation rate), TJC (tender joint count), SJC (swollen joint count), VAS (visual analogue scale), PhGA (physician's global assessment of disease activity), PGA (patient's global assessment of disease activity), and HAQ (Health Assessment Questionnaire). Subjects underwent radiological progression analysis at baseline, which involved frontal X-rays of both hands and wrists. Two radiologists read and analyzed the radiographic images according to the Sharp scoring system revised by van der Heijde. The radiologists had no knowledge of the treatment allocation, the chronology of radiographs, or patients' clinical responses. The Sharp scoring system is revised by van der Heijde, including TSS (total Sharp score), JSN (joint gap narrow score), and JE (joint erosion score). The sum of joint erosion (JE) score and joint space narrowing (JSN) was the value of total Sharp score (TSS). File 2: supplementary materials_data_52w. The supplementary materials are the original data of this study, mainly including the basic information of patients, grouping, disease activity and X-ray sharp score. The basic information of the subjects includes the name, gender, and year of birth. Disease activity was recorded by the researchers at 52 weeks of follow-up, including DAS28 (28-joint count Disease Activity Score), CRP (C-reactive protein), ESR (erythrocyte sedimentation rate), TJC (tender joint count), SJC (swollen joint count), VAS (visual analogue scale), PhGA (physician's global assessment of disease activity), [file 5808400.f1.zip › 5808400.f1/supplementary materials_data_baseline.pdf]

original source data(baseline)

Group	name	gender (1=male, 2=female)	year of birth	Course (month)	DAS28(baseline)	CRP (baseline)	ESR (baseline)	TJC (baseline)	SJC (baseline)	VAS (baseline)	PhGA (baseline)	PGA (baseline)	HAQ(baseline)	TSS(baseline)	JSN(baseline)	JE(baseline)
2	Hyi C	2	1955	13	5.21	19.1	36	6	5	50	60	50	21	21	13	8
2	Yru C	2	1959	6	5.44	35.2	43	4	12	50	60	85	9	18	16	2
1	Fxi C	2	1969	30	8.39	13.81	65	28	24	80	80	80	40	3	2	1
1	Hgu C	1	1968	12	6.84	82.6	24	25	12	60	70	80	15	0	0	0
2	Sh Ch	2	1988	9	6.45	0	36	14	11	65	65	65	16	0	0	0
3	Sho C	1	1964	12	7.35	82.2	86	25	7	50	50	60	15	0	0	0
1	Zli C	2	1981	24	5.17	11.3	21	11	3	50	50	60	29	14	11	3
1	Gxi D	2	1964	24	6	12	83	7	8	45	45	35	18	2	0	2
1	Yli D	2	1973	12	5.12	3.85	25	10	2	50	40	40	8	6	6	0
1	Lxi D	2	1952	30	6.14	15.7	65	12	8	35	30	40	16	20	14	6
3	We Fa	1	1967	60	5.39	13.65	16	11	10	50	90	80	11	71	48	23
2	Eyu F	1	1951	0.5	4.2	0.4	14	11	2	8	6	8	14	13	13	0
3	Pi Fe	2	1950	10	5	9.28	65	4	2	40	50	50	13	3	3	0
1	Lli G	2	1970	240	6.53	5	92	9	9	60	60	60	13	7	5	2
3	Xyu G	2	1955	36	5.81	18.96	46	9	7	50	60	80	25	19	16	3
1	Mxi G	2	1955	30	4.61	6.41	22	5	3	50	50	35	4	14	13	1
3	Jxi G	2	1960	5	4.26	3.42	9	9	3	40	30	40	0	0	0	0
2	Rfe G	2	1957	24	7.71	10.94	28	28	26	70	70	80	15	65	50	15
2	Wqi G	2	1968	16	5.19	1.52	33	4	3	80	45	40	13	0	0	0
3	Jyi H	2	1977	10	3.1	1	14	4	0	10	10	20	2	17	16	1
3	Qru H	2	1956	34	5.62	12	113	3	5	50	50	75	16	96	54	42
3	We Ha	2	1965	4	4.5	32.3	83	1	1	40	40	50	6	14	13	1
2	Jbo H	2	1982	36	3.37	1.49	5	4	4	40	51	51	3	33	23	10
3	Jxi J	2	1964	19	3.48	2.17	12	3	3	20	20	20	0	0	0	0
1	Lli J	2	1980	19	4.43	14.92	23	5	4	30	30	40	3	8	8	0
1	Lu Ji	2	1979	3	6.28	2.7	31	16	11	50	50	50	26	41	14	27
1	Xqi K	2	1954	24	5.48	8.839	68	4	3	65	60	60	14	0	0	0
2	Xqi K	2	1971	16	5.92	8.21	28	11	10	60	72	75	6	36	27	9
1	Csh L	2	1962	24	5.87	17.956	96	4	4	70	50	60	19	0	0	0

Group	name	gender（1=male，2=female ）	year of birth	Course（month）	DAS28(baseline)	CRP（baseline）	ESR（baseline）	TJC（baseline）	SJC（baseline）	VAS（baseline）	PhGA（baseline）	PGA（baseline）	HAQ(baseline)	TSS(baseline)	JSN(baseline)	JE(baseline)
1	Jho L	2	1959	36	4.54	1.12	33	5	1	40	30	30	4	9	7	2
3	Jyu L	2	1996	24	4.77	48.25	26	3	15	30	30	50	1	135	70	65
1	Jpi L	2	1951	1	6.08	14.017	77	11	5	40	45	50	11	1	1	0
1	Sju L	2	1981	7	5.47	6.88	71	3	13	35	50	80	12	5	5	0
3	Xbo L	1	1979	24	6.6	18.6	23	25	5	70	70	70	0	3	2	1
2	Xhu L	2	1972	24	4.15	3	4	10	4	60	60	50	13	12	12	0
3	Fya L	2	1959	7	6.8	45.73	55	14	14	60	60	60	25	1	1	0
3	Ghu L	2	1955	12	6.92	17.2	73	13	13	63	65	63	6	9	8	1
3	Hti L	2	1953	16	4.75	0.03	21	6	6	40	40	55	3	0	0	0
2	Jla L	2	1969	48	5.41	18.5	42	5	3	75	60	50	11	4	4	0
1	Jhu L	2	1977	12	6.5	1.44	22	21	11	60	60	60	40	0	0	0
3	Li Li	2	1969	12	5.52	10.1	43	7	4	60	40	70	7	5	4	1
3	Mi Li	2	1955	30	7.13	12.6	80	18	16	40	40	50	15	40	24	16
2	Syi L	2	1958	8	4.33	8.44	22	4	3	40	50	40	18	1	1	0
2	Xme L	2	1964	24	6.36	19.9	45	10	11	70	70	70	33	68	48	20
2	Wji L	2	1983	36	4.48	2.18	6	8	8	60	60	60	8	74	62	12
2	Bju M	2	1977	12	5.26	3.88	19	12	4	50	50	40	10	10	9	1
1	Gpi M	2	1965	24	6.85	3.32	34	16	13	80	80	85	7	8	8	0
2	Wyi M	2	1988	22	5.74	131.45	80	4	4	70	70	80	17	63	45	18
2	Ji Me	2	1964	48	5.02	36	44	5	3	45	40	30	1	15	14	1
1	Yhu M	2	1974	6	5.25	37.74	63	6	4	30	60	55	10	5	5	0
1	Xro N	2	1978	72	7.35	2.87	40	26	11	70	70	80	20	4	2	2
3	Yli P	2	1962	30	6.19	11.2	71	10	6	53	47	50	5	2	1	1
3	Jju R	2	1972	24	4.17	3.28	10	6	3	50	40	60	2	50	33	17
3	Yli S	1	1954	12	5.89	27.3	52	7	8	60	70	70	32	7	7	0
3	Cxi S	2	1972	1	6.8	4.9	41	21	8	60	60	80	13	0	0	0
1	Syu S	2	1953	20	5.66	15.6	113	6	3	35	50	100	14	3	3	0
2	Xla S	2	1955	34	7.53		48	26	16	60	60	50	21	3	1	2
3	Mpi T	2	1956	34	6.83	1.05	25	23	14	60	60	60	27	54	18	36
3	Ji Ti	2	1958	20	6.24	8.93	32	14	13	50	40	40	27	12	6	6

Group	name	gender（1=male, 2=female ）	year of birth	Course（month）	DAS28(baseline)	CRP（baseline）	ESR（baseline）	TJC（baseline）	SJC（baseline）	VAS（baseline）	PhGA（baseline）	PGA（baseline）	HAQ(baseline)	TSS(baseline)	JSN(baseline)	JE(baseline)
2	Xya T	2	1976	36	4.8	7.5	28	6	2	50	40	60	13	73	54	19
2	Sde W	1	1968	32	6.4	112	113	4	20	50	80	50	10	35	21	14
2	Hqi W	2	1963	36	6.96	27.7	87	17	6	60	70	70	9	0	0	0
2	Jyu W	1	1953	36	6.17	52.1	50	12	8	50	70	80	10	22	13	9
2	Li Wa	2	1982	15	4.68	3.45	10	9	6	50	50	60	22	5	5	0
1	Lju W	2	1975	15	5.37	7.79	55	6	6	36	47	53	3	12	12	0
1	Mi Wa	2	1982	7	5.19	9.25	20	7	3	80	70	30	13	6	6	0
3	Pi Wa	1	1959	12	4.46	3.55	30	4	2	40	40	40	18	0	0	0
3	Rha W	1	1963	72	6.01	0.68	30	12	9	60	60	60	7	0	0	0
2	Sli W	2	1957	36	6.31	17.4	31	16	6	70	70	70	0	2	2	0
2	Sfe W	2	1954	24	5.98	35	21	14	14	50	50	50	27	5	2	3
1	Szh W	2	1962	72	4.81	9.86	35	4	4	45	60	60	3	13	9	4
3	Xxi W	2	1958	24	4.71	3.45	16	8	5	40	50	50	19	1	0	1
1	Xho W	2	1970	12	4.17	20.1	17	3	3	52	51	51	5	0	0	0
1	Xyi W	2	1955	6	6.35	82.06	82	10	10	43	45	54	17	5	4	1
2	Yju W	1	1973	6	6.2	21.6	40	15	10	40	50	50	4	0	0	0
2	Cli W	1	1981	24	6.47	22.3	64	11	11	55	80	100	20	2	2	0
3	Sju W	2	1959	12	4.59	15.8	33	1	21	20	30	20	2	10	8	2
2	We We	2	1953	240	6.47	6	51	12	11	60	60	60	9	8	7	1
3	Gue W	2	1959	13	4.93	7.9	44	8	0	50	50	50	8	6	6	0
2	Yme W	2	1982	18	5.22	1.1	26	9	5	45	45	45	9	0	0	0
2	Xme W	2	1965	26	5.18	3.08	25	7	7	50	50	60	7	3	0	3
1	Ypi X	2	1969	1	4.31	1.01	11	8	3	40	30	40	0	0	0	0
1	Hya X	2	1977	19	6.36	29.4	28	15	13	60	60	50	14	7	7	0
3	Swe X	2	1983	3	4.51	2.03	21	9	1	30	30	30	1	2	2	0
2	Hni Y	1	1972	6	5.71	16.83	20	12	12	50	45	45	6	10	8	2
1	Ju Ya	2	1949	5	4.7	20.3	29	4	8	30	60	60	9	8	8	0
2	Li Ya	2	1985	9	5.32	4.67	39	11	3	30	30	30	1	1	1	0
3	Lxi Y	2	1970	24	5.08	25.02	26	5	5	65	80	80	9	10	9	1
3	Xyu Y	2	1973	6	3.91	1.37	6	6	6	42	39	38	4	0	0	0

Group	name	gender（1=male, 2=female ）	year of birth	Course（month ）	DAS28(baseline ）	CRP （baseline）	ESR （baseline）	TJC （baseline）	SJC （baseline）	VAS （baseline）	PhGA （baseline）	PGA （baseline）	HAQ(baseline)	TSS(baseline)	JSN(baseline)	JE(baseline)
2	Xne Y	2	1966	6	3.94	3.28	8	8	3	30	40	40	3	6	5	1
3	Hxi Z	2	1956	24	3.51	11.6	6.1	3	4	50	60	90	14	12	9	3
3	Chu Z	2	1968	12	5.56	4.11	16	13	4	74	80	50	33	5	5	0
1	Fyi Z	2	1966	36	6.1	6.71	41	10	10	60	70	60	16	7	7	0
3	Jhu Z	2	1970	24	4.38	9.98	11	6	5	50	40	50	7	12	8	4
3	Jqi Z	2	1960	4	6.36	30	92	6	6	80	80	80	9	1	0	1
1	Qni Z	2	1979	9	5.93	2.08	22	16	6	60	60	60	4	0	0	0
1	Xya Z	2	1982	26	5.07	5.29	18	11	5	40	40	40	3	26	12	14
2	Xzh Z	2	1958	28	6.32	3.28	23	13	12	80	70	90	18	56	40	16
2	Zch Z	1	1963	8	5.37	55.7	33	13	3	30	40	30	1	10	7	3
2	Mju Z	2	1984	9	5.64	12.2	46	10	3	50	40	50	9	0	0	0
3	Zyi Z	2	1987	18	5.12	11.5	25	5	5	70	70	70	22	23	20	3
3	Cne Z	2	1960	3	7.56	3.69	50	28	7	80	90	100	33	0	0	0
1	Mfa Z	2	1952	12	7.08	8.11	18	28	12	80	80	80	25	0	0	0
3	Mi Zh	2	1969	36	5.38	1.88	16	8	8	75	84	77	8	39	27	12
1	Jme Z	2	1965	13	4.49	12.37	13	6	4	54	60	50	4	14	11	3
2	Rqi H	2	1971	56	5.39	1	23	10	8	45	50	35	6			
3	Xyu W	2	1967	60	5.47	26.77	75	3	3	70	70	70	8			
3	Ju Wa	2	1978	16	5.59	1	26	10	5	65	60	45	9			
1	Sju Z	2	1957	36	5.45	32	88	4	3	50	50	70	11			
2	Lli H	2	1981	68	7.16	15	50	15	16	80	70	60	7			
1	Yi Wa	2	1967	4	5.91	4	19	17	9	50	55	45	19			
3	Ypi M	2	1963	29	4.94	5	34	3	8	50	40	50	4			
3	Hyu X	2	1957	1	6.69	1.36	54	14	7	75	60	80	19			
3	Yme Z	2	1979	41	5.98	5.15	26	15	6	60	60	80	6			
2	Fro G	2	1952	14	7.02	8	22	20	15	90	80	80	18			
1	Sxi R	2	1966	48	7.59	72.34	91	17	11	85	85	85	34			
3	Xi Ya	2	1968	12	5.38	26.05	77	2	5	65	60	70	6			
2	Xla Q	2	1963	49	4.27	1.93	23	4	2	40	30	30	5			
1	Jli C	2	1964	34	6.65	59.89	96	8	7	80	70	70	30			

Group	name	gender （1=male, 2=female ）	year of birth	Course （month ）	DAS28(baseline)	CRP （baseline）	ESR （baseline）	TJC （baseline）	SJC （baseline）	VAS （baseline）	PhGA （baseline）	PGA （baseline）	HAQ(baseline)	TSS(baseline)	JSN(baseline)	JE(baseline)
2	Jji Z	2	1971	4	5.5	1	23	11	7	50	40	70	15			
2	Xwa L	2	1974	36	3.9	8.1	18	3	3	30	30	40	1			
3	Lju M	2	1954	28	7.16	9.09	45	16	16	80	80	50	10			
2	Zyu L	1	1973	60	4.83	53	30	4	3	60	50	80	13			
1	Ju Wa	2	1967	6	5.89	9.4	23	15	6	60	60	60	4			
1	Cfa Z	1	1949	5	7.68	14	86	15	16	90	80	90	43			
3	Wxi Y	1	1964	5	5.32	3.56	38	5	7	55	50	80	7			
1	Ywe L	2	1983	24	6.14	160	104	7	4	60	70	80	15			
3	Szh Y	2	1951	4	8.38	46	96	25	20	80	70	80	40			
1	Lpi L	1	1953	14	4.57	10.22	68	2	2	30	40	30	0			
2	Zlo Y	1	1964	36	5.66	53.24	66	3	3	90	60	50	5			
2	Xta P	2	1985	8	5.73	1	36	10	10	40	40	20	0			
1	Dzh Y	2	1975	18	5.9	14.42	49	7	9	60	60	50	15			
1	Lru Z	2	1962	15	4.06	7	23	4	2	25	30	30	1			
3	Wdo L	1	1971	30	3.95	12.27	6	7	8	30	40	60	0			
2	Li Ma	2	1964	60	4.93	20	23	5	4	65	50	50	11			
1	Gya L	1	1990	120	7.18	100.88	65	16	10	80	80	80	23			
3	Hzh Z	1	1983	24	4.61	17	13	7	5	50	30	20	0			
1	Dfa Y	2	1950	12	6.23	4.51	64	8	7	70	60	70	15			
1	Yzh B	2	1962	36	5.62	12.21	25	10	10	50	60	50	13			
3	Zpi S	2	1963	4	4.68	1.15	44	4	3	30	30	35	2			
1	Cta D	2	1986	48	6.47	8.13	33	15	13	60	70	60	9			
2	Yqi Z	2	1956	30	4.67	20.43	46	2	3	50	40	50	11			
1	Mxi X	2	1979	36	5.89	7.77	27	9	16	55	60	50	17			
3	Cla A	2	1961	28	7.54	7.71	69	19	13	80	75	80	31			
2	Ju Wa	2	1966	120	6.65	16.74	52	14	13	55	60	65	14			
3	Dda D	2	1984	9	7.87	20.85	49	27	20	70	79	70	23			
3	Fme L	2	1971	36	4.77	6.64	25	5	4	50	40	50	9			
1	Jhu C	2	1965	46	6.3	26	17	16	24	50	70	70	29			
3	Ji Zh	2	1970	24	8.3	83.8	86	23	17	95	80	85	30			

Group	name	gender（1=male, 2=female ）	year of birth	Course（month）	DAS28(baseline)	CRP（baseline）	ESR（baseline）	TJC（baseline）	SJC（baseline）	VAS（baseline）	PhGA（baseline）	PGA（baseline）	HAQ(baseline)	TSS(baseline)	JSN(baseline)	JE(baseline)
2	Qso L	1	1975	28	6.91	80.94	72	13	14	60	60	80	19			
3	Fqi W	2	1976	72	7.03	1	31	18	16	80	75	85	23			
3	Lfe W	2	1956	12	6.19	18.94	77	9	5	60	60	80	20			
2	Xme L	2	1975	48	5.38	11.76	27	9	6	50	50	45	6			

Note：DAS28 28-joint count Disease Activity Score,CRP C-reactive protein,ESR erythrocyte sedimentation rate,TJC tender joint count, SJC swollen joint count,VAS visual analogue scale,PhGA Physician’s global assessment of disease activity,PGA Patient’s global assessment of disease activity,HAQ Health Assessment Questionnaire,TSS total Sharp score,JSN joint gap narrow score,JE joint erosion score.
